# Cross-species urinary metabolomics identifies 2-hydroxyisovalerate as a candidate biomarker for mtDNA-based disorders

**DOI:** 10.1007/s11306-026-02518-1

**Published:** 2026-08-20

**Authors:** Marianne Venter, Karin Terburgh, Jeremie Z. Lindeque, Emi Ogasawara, Mirian C. H. Janssen, Jan A. M. Smeitink, Kazuto Nakada, Roan Louw

**Affiliations:** 1 Mitochondria Research Group, Biomedical and Molecular Metabolism Research (BioMMet), North-West University, Potchefstroom, South Africa; 2 Department of Biological Sciences, Graduate School of Science, Osaka University, Toyonaka, Osaka 560-0043 Japan; 3 Department of Pediatrics, Radboud Center for Mitochondrial Medicine, Radboud University Medical Center, Nijmegen, Netherlands; 4 Department of Internal Medicine, Radboud Center for Mitochondrial Medicine, Radboud University Medical Center, Nijmegen, Netherlands; 5 Institute of Life and Environmental Sciences, University of Tsukuba, Tsukuba, Japan

**Keywords:** MtDNA, Heteroplasmy, Mitochondrial disease, Mito-mice, M.3243A > G, Metabolomics, 2-Hydroxyisovalerate

## Abstract

Pathogenic mitochondrial DNA (mtDNA) mutations contribute to a broad spectrum of both common and rare metabolic diseases. However, clinical presentation is highly variable and only partially explained by the proportion of mutant mtDNA or heteroplasmy. With the relationship between mutation burden and clinical manifestation poorly defined, controlled models are required to uncover underlying mechanisms. Here, we explore the metabolic consequences of increasing heteroplasmy in a well-characterised mouse model harbouring a pathogenic mtDNA deletion. Untargeted urinary metabolomics reveals distinct mutation load-dependent metabolic shifts with some metabolites declining early on, while others exhibit threshold-like increases beyond ~ 60% mutation load — the level at which lactic acidemia and OXPHOS defects become apparent in this model. To assess translational relevance, we examined these heteroplasmy-associated metabolites in urine from patients carrying the most common mtDNA mutation, m.3243 A > G. Several of these metabolites were differentially expressed in patients relative to controls, with conserved directionality across species. Among these, 2-hydroxyisovalerate (2-HIVA), which was most strongly affected in the mouse model, also emerged as the top discriminator in patients. Receiver operating characteristic analysis indicated that urinary 2-HIVA has strong discriminatory power, supporting its potential utility as a biomarker for mtDNA-based disorders. These findings enhance our understanding of mtDNA-related disease pathophysiology and establish a foundation for further validation studies.

**Graphical abstract:**

**Figure:**
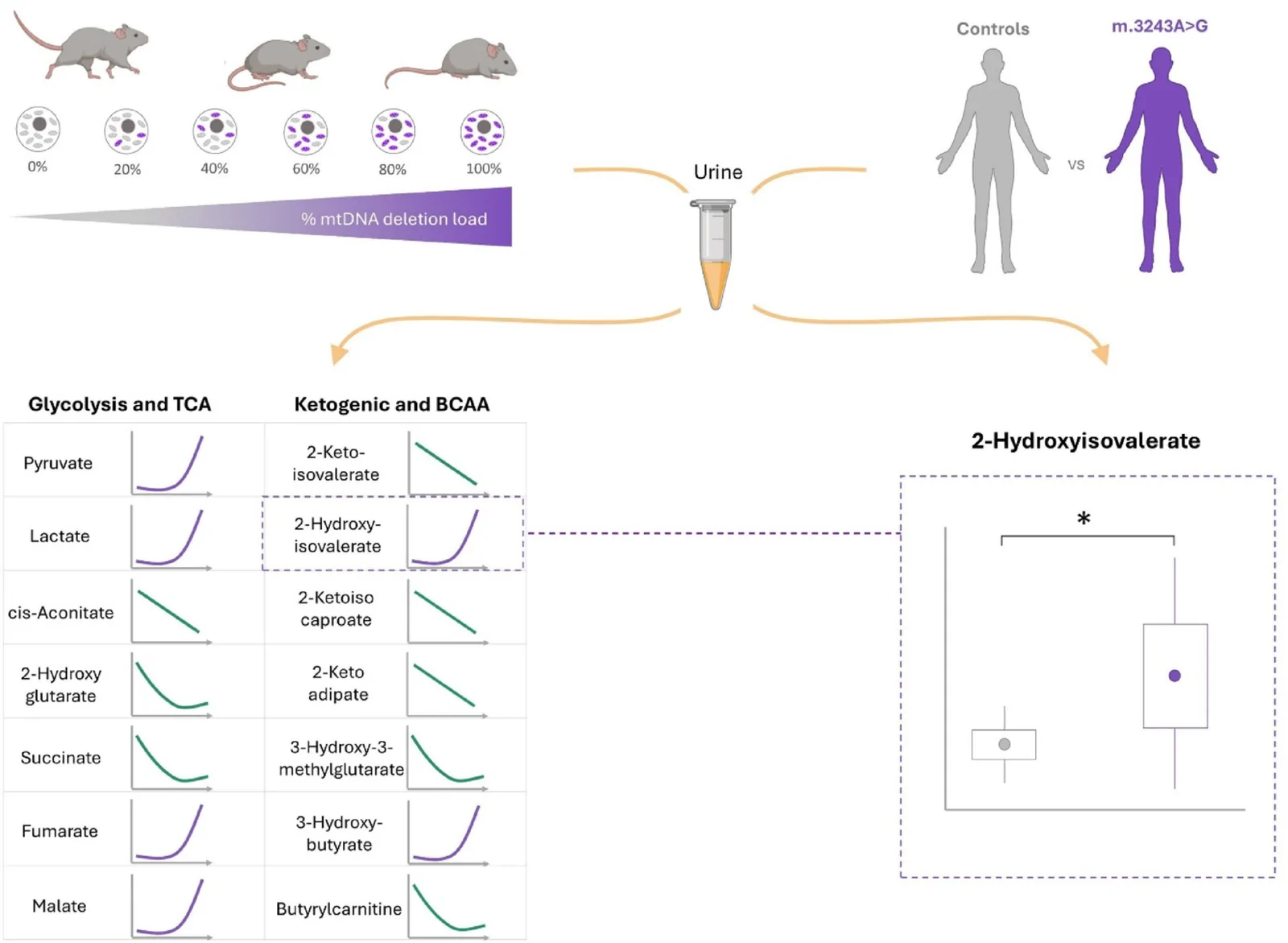

**Supplementary Information:**

The online version contains supplementary material available at https://doi.org/10.1007/s11306-026-02518-1.

## Highlights

mtDNA mutation load in mice correlates with progressive metabolite shifts.

~60% heteroplasmy triggers OXPHOS-deficiency linked metabolic alterations in mice.

Distinct mtDNA mutations in mice and humans cause shared urinary metabolite changes.

2-Hydroxyisovalerate is strongly perturbed in both Mito-mice and m.3243A>G patients.

Urinary 2-HIVA is a promising biomarker candidate for mtDNA-based disorders.

**Supplementary Information:**

The online version contains supplementary material available at https://doi.org/10.1007/s11306-026-02518-1.

## Introduction

Mitochondrial dysfunction, resulting from mutations in mitochondrial DNA (mtDNA), not only accounts for most primary mitochondrial disorders (Gorman et al., 2015) but also contributes to several common age-related and metabolic disorders, including diabetes, cardiovascular disease, neurodegenerative diseases, and cancer (Thorburn, 2004; Keshavan & Rahman, 2018; Parikh et al., 2019).

Owing to the multicopy nature of mitochondria within the cell and of mtDNA within the mitochondrion, mutant and wildtype mtDNA copies can coexist in a state called heteroplasmy (Wallace & Chalkia, 2013; Chinnery & Hudson, 2013; Taylor & Turnbull, 2005). However, the clinical expression of mtDNA mutations is only partially explained by heteroplasmy and is highly variable with many mutations showing poor genotype–phenotype correlation. Similar clinical phenotypes can arise from distinct mutations, while a single mutation may give rise to a wide spectrum of symptoms (Morgan-Hughes et al., 1995; Craven et al., 2017).

The latter is seen in the most prevalent pathogenic mtDNA mutation found in humans ― the m.3243 A > G point mutation in the *MT-TL1* gene, encoding mt-tRNA^Leu (UUR)^ (Gorman et al., 2015; Manwaring et al., 2007; Elliott et al., 2008). This single mtDNA mutation has a wide variety of manifestations that range greatly in severity and include isolated or concomitant non-syndromic symptoms, such as myopathy, cardiomyopathy and peripheral neuropathy, as well as clearly defined syndromes, such as mitochondrial encephalomyopathy, lactic acidosis and stroke-like episodes (MELAS) or maternally inherited diabetes and deafness (MIDD) (Shen & Du, 2021).

In patients carrying the m.3243 A > G mutation, heteroplasmy, age, and mtDNA copy number together explain only ~ 40% of the variance in disease severity (Grady et al., 2018), as assessed by the Newcastle Mitochondrial Disease Adult Scale (NMDAS) — a validated tool for quantifying disease burden and progression in mitochondrial disorders (Schaefer et al., 2006). Hence, in humans, mtDNA mutation-related disease penetrance and clinical presentation are likely to be influenced by other factors such as the environment, nuclear genetics, epigenetics, and medication (Grady et al., 2018; Pickett et al., 2018). Given these confounding conditions, mechanistic links between mutation burden and clinical outcome are obscured. To disentangle these complexities, the use of higher-order animal models, harbouring pathogenic mtDNA mutations remains essential. Although the development of such animal models have historically been limited by technical challenges (Dunn et al., 2012), recent advances in mtDNA editing is paving the way for an expanding repertoire of mtDNA disease models (Barrera-Paez et al., 2025; Qi et al., 2021; Lee et al., 2021).

The first mouse model developed to study the effect of mtDNA heteroplasmy is the Mito-mice model, which carries a single large 4696-bp mtDNA deletion that affects six mt-tRNAs (tRNA^Lys^, tRNA^Gly^, tRNA^Arg^, tRNA^Leu^, tRNA^Ser^, and tRNA^His^) and seven subunits of oxidative phosphorylation (OXPHOS) complexes (four in CI, one in CIV, and two in CV) (Inoue et al., 2000). This model offers the opportunity to study the graded biochemical effects of increasing heteroplasmy under defined genetic and environmental conditions as these animals exhibit a well-defined, mutation load-dependent phenotype. At mutation loads below 50% deleted mtDNA (ΔmtDNA), no detectable OXPHOS defects or clinical symptoms are observed, making these animals suitable as controls. The earliest clinical manifestation — lactic acidemia — emerges at mutation loads exceeding 50% (Nakada et al., 2008; Ogasawara et al., 2010; Tanaka et al., 2008). As heteroplasmy surpasses 60%, OXPHOS defects appear, accompanied by the expression of memory impairment phenotypes (Tanaka et al., 2008; Nakada & Hayashi, 2011). Ultimately, mutation loads > 75% result in the onset of disease phenotypes such as significant lactic acidemia, low body weight, sperm abnormalities, hearing impairment, systemic ischaemia, atrioventricular block, and renal failure (Inoue et al., 2000; Nakada et al., 2008; Nakada et al., 2006; Nakada et al., 2004).

Despite this extensive characterisation of Mito-mice, their metabolome remains largely unexplored. Metabolomic profiling has emerged as a valuable approach to understanding mitochondrial pathophysiology, revealing disruptions in amino acid metabolism, redox balance, and tricarboxylic acid (TCA) cycle function across multiple disease contexts (Esterhuizen et al., 2017). Urine, as a non-invasive and systemically reflective biofluid, offers valuable insight into the organism-wide metabolic consequences of mitochondrial dysfunction. However, systematic investigations into how progressive increases in mtDNA mutation load affect the metabolome remain scarce — particularly in well-controlled in vivo models.

In this study, we applied urinary metabolomics to characterise systemic metabolic alterations associated with increasing levels of mtDNA heteroplasmy, and thus OXPHOS deficiency, in Mito-mice. To assess the relevance of these findings in a human context, we further examined selected metabolites in adults carrying the m.3243 A > G mutation, diagnosed with classic MELAS or MIDD. This approach enabled a unique view of more nuanced metabolic alterations associated with increasing mutation load and OXPHOS dysfunction, revealed conserved metabolic disturbances across species, and identified 2-hydroxyisovalerate (2-HIVA) — a branched-chain amino acid catabolite — as a translatable candidate biomarker of mtDNA-based disorders.

## Methods

Urinary metabolomes were analysed using a multi-platform approach, including untargeted gas chromatography time-of-flight mass spectrometry (GC–TOF–MS) and targeted liquid chromatography tandem mass spectrometry (LC–MS/MS). Mouse urine samples were newly collected and analysed for this study, whereas human urine data were reprocessed from a previously published dataset (Esterhuizen et al., 2021) using stricter inclusion criteria and targeted re-extraction of metabolites from the original untargeted data.

### Animals and sampling

Male (*n* = 23) and female (*n* = 9) Mito-mice of varying age (6–16 months) were used for this study. The animals were generated by introducing ΔmtDNA from cultivated cells into B6 strain mouse zygotes (F0) via cell fusion techniques as reported previously (Inoue et al., 2000; Nakada et al., 2004). A combination of random genetic drift and selection during oocyte development leads to F1 – F3 mice carrying varying ΔmtDNA heteroplasmy levels at birth. Since the generation of these animals is challenging, the number of mice included in the study was based on available live animals at the time of sample collection. Mice were housed at the University of Tsukuba, Japan, under controlled environmental conditions with *ad libitum* access to food and water. All procedures were approved by the Institutional Animal Care and Use Committee of the University of Tsukuba (No. 22–328).

In the Mito-mice model, ΔmtDNA loads are approximately uniform throughout most tissues of individual mice and tail snip loads are an established indicator of tissue mutation loads (Inoue et al., 2000; Nakada et al., 2001). Tail snip ΔmtDNA levels were determined using real-time polymerase chain reaction as previously described (Sato et al., 2005). Spot urine samples were collected from non-fasting mice (*n* = 32) with tail snip mtDNA mutation loads ranging from 0 to 80%. After collection, urine samples were deproteinised with acetonitrile and dried before transfer to South Africa, where they were stored at − 80 °C until further use.

### Human subjects and sampling

Urine metabolomics data were re-evaluated from a previously published cohort (Esterhuizen et al., 2021), with the current analysis restricted to adult patients (age > 21 years) carrying a confirmed m.3243 A > G mutation and meeting the diagnostic criteria for classic MELAS or MIDD (Table 1), in order to reduce clinical and metabolic heterogeneity. Urine samples were collected from patients and healthy controls at the Radboud Centre for Mitochondrial Medicine, Nijmegen, the Netherlands. All participants provided written informed consent for the use of their samples in research. Although treatment with medication and/or supplementation in patients could not be suspended, the following exclusion criteria were applied to improve sample homogeneity: (i) a prior medical diagnosis in control participants; (ii) medication or supplement use by controls at the time of sample collection; and (iii) history of organ transplantation among patients. Ethical approval for the study was granted by the Health Research Ethics Committee (HREC) of North-West University, South Africa (REC-130913-037; NWU-00170-13-S1), and the Ethics Committee of the Nijmegen-Arnhem region in the Netherlands (2010/183; NL32683.091.10). All procedures were conducted in accordance with institutional guidelines and the principles of the Declaration of Helsinki (1975; revised 2013).

Urine samples were collected under standardised conditions and stored at − 80 °C until further use. Mutation loads of the m.3243 A > G variant was quantified in urinary epithelial cells using polymerase chain reaction, restriction enzyme digestion, and pyrosequencing as previously described (Esterhuizen et al., 2021). Disease severity and progression were assessed using the Newcastle Mitochondrial Disease Adult Scale (NMDAS) (Schaefer et al., 2006). Urinary osmolality was determined by Ampath Laboratories (South Africa) using an Advanced Osmometer (IEPSA Diagnostics, South Africa).

**Table 1:** Demographic and clinical characteristics of control participants and patients with the m.3243 A > G mutation

Characteristics	Controls	m.3243 A > G
Total, n	21	36
Age (years)	43.1 (21–66)	47.0 (22–65)
Sex (F = female, M = male)	F = 13, M = 8	F = 23, M = 13
m.3243 A > G mutation load in urinary epithelial cells	–	62.2 (30–97)
NMDAS score	–	26.3 (5–82)
Clinical subtypes	–	MELAS = 7, MIDD = 29
Insulin treatment	–	Yes = 24, No = 12
Anti-epileptic treatment	–	Yes = 7, No = 29

Values are presented as means with ranges in parentheses where applicable. NMDAS = Newcastle mitochondrial disease adult scale. MIDD = maternally inherited diabetes and deafness. MELAS = mitochondrial encephalomyopathy, lactic acidosis, and stroke-like episodes

### Sample preparation for metabolic profiling

For mouse samples, the dried urine pellets obtained after deproteinisation with acetonitrile (Sect. 2.1) were first reconstituted in water prior to metabolite extraction and analysis. Human urine samples were analysed directly from frozen aliquots following thawing. The urine samples were prepared and analysed according to methods previously described for mouse (Terburgh et al., 2021) and human (Esterhuizen et al., 2021) samples with methodological differences between species reflecting species-specific protocol optimisation and independent analytical workflows. Urinary creatinine concentrations were determined using the QuantiChrom™ Creatinine Assay Kit (BioAssay Systems). For each analytical platform, the volume of urine corresponding to a fixed creatinine content (0.0625 µmol for mouse urine and 0.25 µmol for human urine) was calculated and used for sample preparation. This approach standardises urine concentration before extraction and has previously been shown (Esterhuizen et al., 2021; Esterhuizen et al., 2019) to improve analytical repeatability across samples with varying urinary concentrations. For mouse and human urine respectively, a quality control (QC) sample was prepared from pooled aliquots of all samples. Internal standards were added to each sample as follows: 3-phenylbutyric acid (100 ppm), norleucine (50 ppm) and tropic acid (50 ppm) to mouse urine for GC–TOF–MS; 3-phenylbutyric acid (50 ppm) to human urine for GC–TOF–MS and a mixture of deuterated amino acid and acylcarnitine isotopes to mouse urine (2.5 ppm) and human urine (10 ppm) for LC–MS/MS. Thereafter, samples were evaporated under a stream of nitrogen gas at 37˚C and derivatised prior to their analysis. GC–TOF–MS samples were oximated using methoxyamine hydrochloride (20 mg/mL in pyridine) and subsequently silylated using *N*,*O*-bis(trimethylsilyl) trifluoroacetamide: trimethylchlorosilane (99:1, v/v) with each reaction incubated for 60 min at 60 °C. LC–MS/MS samples were butylated using 1-butanol: acetyl chloride (4:1,v/v) with incubation for 15 min at 65 °C (for mouse urine) or 60 min at 50 °C (for human urine). Samples were then evaporated to dryness under a stream of nitrogen gas at 37 °C and reconstituted in water: acetonitrile (50:50, v/v).

### GC–TOF–MS analysis

Samples were analysed on a Leco Pegasus HT GC-TOF-MS system with Agilent 7890 A GC front-end. Chromatography was performed on a Restek Rxi-5Sil MS capillary column (mouse urine: 28.6 m x 250 μm x 0.25 μm; human urine:10 m x 200 μm x 0.18 μm) using the GC oven temperature programs provided in Table S1. The front inlet temperature was kept at 250 °C and helium was used as carrier gas at a constant flow rate of 1.5 mL/min. A sample volume of 1 µL was injected with the injector operating in split mode (1:10 ratio). Eluting compounds were subjected to electron impact ionisation (70 eV) with the transfer line and ion source temperatures held at 225 °C and 200 °C, respectively. Mass spectra within the scan range of 50–950 m/z were acquired at a scan rate of 20 spectra/second. Peaks with 5 apexing masses were detected using an expected peak width of 3 s and a signal-to-noise ratio of > 20. Data was acquired using ChromaTOF software (v4.5x, LECO Corporation) and included automatic baseline correction using the “spanning” method (offset set to 1, positioned just above the noise level) and automatic signal smoothing. For mouse urine samples, untargeted data extraction and peak alignment were conducted using the Statistical Compare feature in ChromaTOF, which aligns peaks across samples based on retention time and mass spectral similarity. In contrast, selected metabolites were re-extracted from raw human urine data using MET-IDEA (metabolomics ion-based data extraction algorithm) (Broeckling et al., 2006). MET-IDEA enables refined ion-based peak detection and integration, improving consistency and precision of quantification by reducing peak misalignment and minimising missing values. All extracted data were manually reviewed to confirm accurate peak boundaries and integration quality.

### LC–MS/MS analysis

Samples were analysed on an Agilent 6410 LC-MS/MS system with 1200 series LC front-end. Chromatography was performed on an Agilent C18 Zorbax SB-Aq reverse phase column (2.1 mm x 150 mm x 3.5 μm) according to the gradient-elution programs given in Table S2. The column temperature was kept at 35 °C, with water and acetonitrile (each containing 0.1% formic acid) used as mobile phases. A sample volume of 4 µL (mouse urine) or 5 µL (human urine) was injected. Eluting compounds were subjected to positive electrospray ionisation under the following source conditions: capillary voltage of 3 500 V; nitrogen source gas at 300 °C with a 7.5 L/min flow rate; and a nebuliser pressure of 30 psi. Spectra were acquired using the multiple reaction monitoring mode for specific amino acid and acylcarnitines (Table S3) at an electron multiplier voltage of 300 V. Data acquisition and extraction was done using Agilent’s MassHunter Workstation software (Data acquisition for 6400 Series Triple Quadrupole, v B02.01; Qualitative Analysis and Quantitative Analysis software, v B06.00).

### Data processing

The spectral data obtained from each analytical platform were individually processed in Excel (Microsoft 365). Urine data were normalised to platform-specific internal standards, with human data additionally normalised to osmolality. A missing value filter was applied to remove features from the datasets that were not detected in 80% of at least one human sample group. Next, features with a coefficient of variation greater than 50% in QC samples were removed from the datasets, with an additional Fisher’s ratio (< 0.05) noise filter and missing value replacement (half of the minimum value) applied to human urine datasets. Thereafter, the data were transformed (mouse: cubic root; human: logarithmic) and pareto scaled in MetaboAnalyst v.6.0 (Pang et al., 2024). For the GC-MS data, metabolite identities were assigned to features by electron ionisation mass spectral and retention time matching using commercial (NIST11) and in-house libraries, together with public databases (Table S4). Metabolites detected by LC-MS/MS were identified using the targeted analytical method based on authentic standards, retention times and MRM transitions.

### Statistical analyses

Correlations between urinary metabolite levels and mtDNA mutation load and/or NMDAS scores were assessed in MetaboAnalyst using simple Pearson and partial Pearson correlations, adjusted for age and sex. A correlation coefficient (r) > 0.4 — generally indicative of a moderate to strong relationship — and a false discovery rate (FDR)-adjusted *p* < 0.05 were considered statistically significant. To evaluate the robustness of observed associations and account for non-linear relationships, Spearman’s rank correlations were also computed as selected metabolites showed threshold or quadratic-like behaviour even after data transformation and scaling. These results were consistent with Pearson’s correlations (see Supplementary Table S5), supporting the reliability of the observed patterns.

Differential urinary metabolite expression between m.3243 A > G patients and healthy controls was assessed using MetaboAnalyst’s implementation of the limma (linear models for microarray data) package (Pang et al., 2022; Ritchie et al., 2015; Phipson et al., 2016). Limma is well-suited for small-sample metabolomics studies as it fits linear models to each metabolite and applies an empirical Bayes method to stabilise variance estimates across features, enhancing statistical power and reliability. This approach improves sensitivity for detecting true differences, reduces false positives, and accommodates covariates, making it a robust alternative to traditional univariate tests (Pang et al., 2022; Ritchie et al., 2015; Phipson et al., 2016). Analyses were performed both unadjusted and adjusted for age and sex. P-values were corrected for multiple comparisons, with statistical significance defined as an FDR-adjusted *p* < 0.05. Effect sizes were calculated from unscaled log-transformed data to quantify the magnitude of group differences, with Cohen’s d ≥ 0.8 interpreted as a large effect.

To further assess biomarker potential, multivariate and univariate receiver operating characteristic (ROC) analysis were conducted in MetaboAnalyst. For multivariate models, a support vector machine (SVM) classifier with SVM-based internal feature ranking was used. Model performance was evaluated using Monte Carlo cross-validation with balanced subsampling: in each iteration, two-thirds of samples were randomly assigned to training (including feature selection), and one-third to testing. This process was repeated 500 times to estimate average area under the curve (AUC) and derive 95% confidence intervals from the distribution of test results. For univariate ROC analyses, individual metabolites were assessed for their standalone discriminatory power. AUC values and 95% confidence intervals were calculated using 500 bootstrapping resamples. Optimal diagnostic cutoffs were determined using the “closest to top-left corner” criterion. ROC AUC values were used to evaluate the discriminatory performance, with AUC ≥ 0.8 indicating good classification performance.

## Results

### Increasing mutation load correlates with a progressive metabolic shift in Mito-mice

To investigate the metabolic consequences of increasing mtDNA mutation burden, correlations between urinary metabolite levels and mutation load were evaluated in Mito-mice (*n* = 32) using both unadjusted and covariate-adjusted models. Metabolite ratios indicative of redox state, tricarboxylic acid (TCA) cycle activity, and amino acid metabolism were also assessed.

**Fig. 1:**
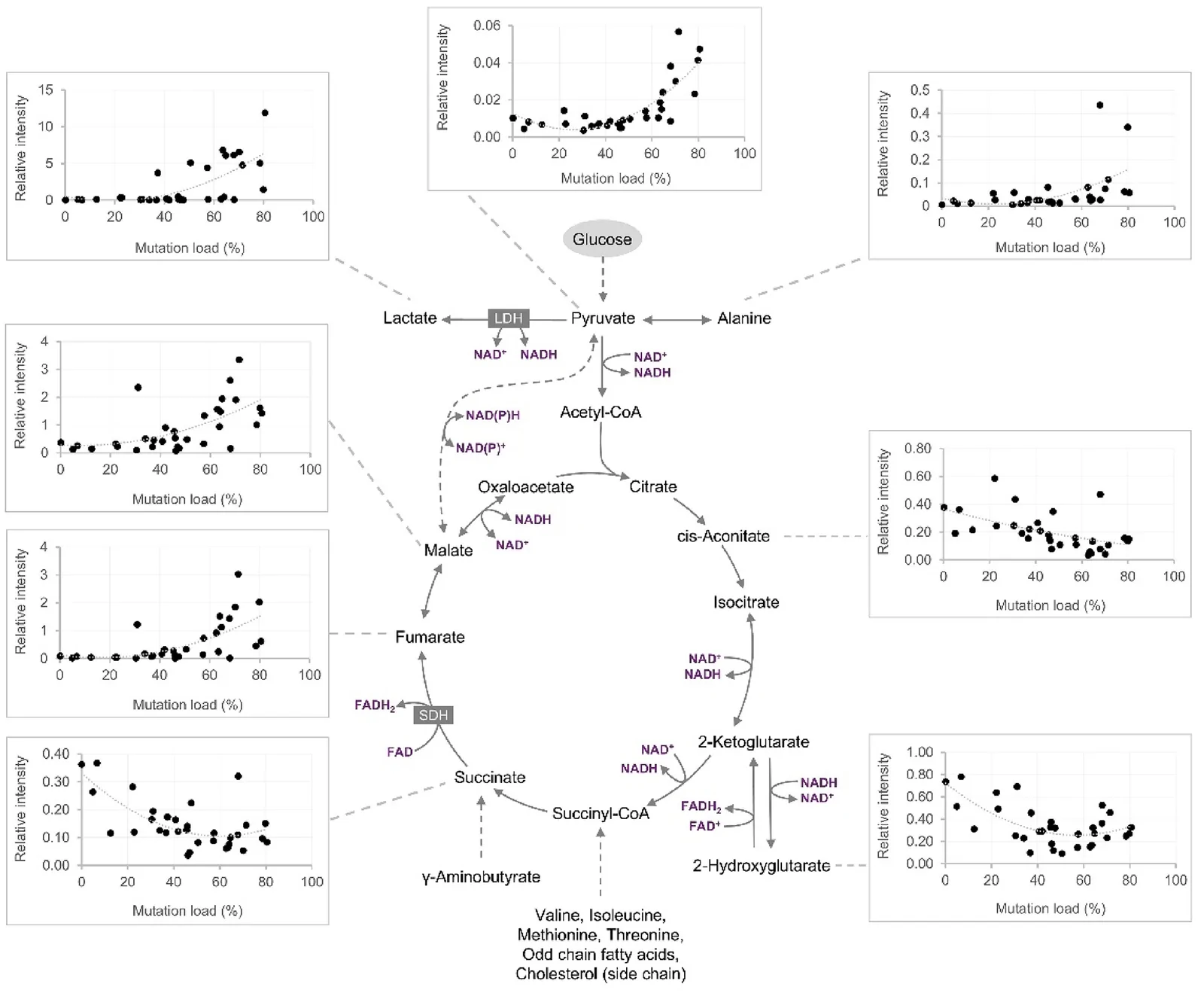
Glycolytic and TCA cycle intermediates correlate with mtDNA mutation load in Mito-mice. Scatterplots depict relative intensities (y-axis) of urinary metabolites in individual Mito-mice (*n* = 32) with varying levels of mtDNA mutation loads (x-axis). Of those detected, only metabolites that significantly correlated with mutation load are displayed in scatterplots (Pearson’s *r* > 0.4; FDR-*p* < 0.05). Acetyl-CoA, succinyl-CoA, oxaloacetate and isocitrate were not detected. Solid and dashed arrows represent single- and multi-step reactions. Abbreviations: FDR, false discovery rate; LDH, lactate dehydrogenase; SDH, succinate dehydrogenase

**Fig. 2:**
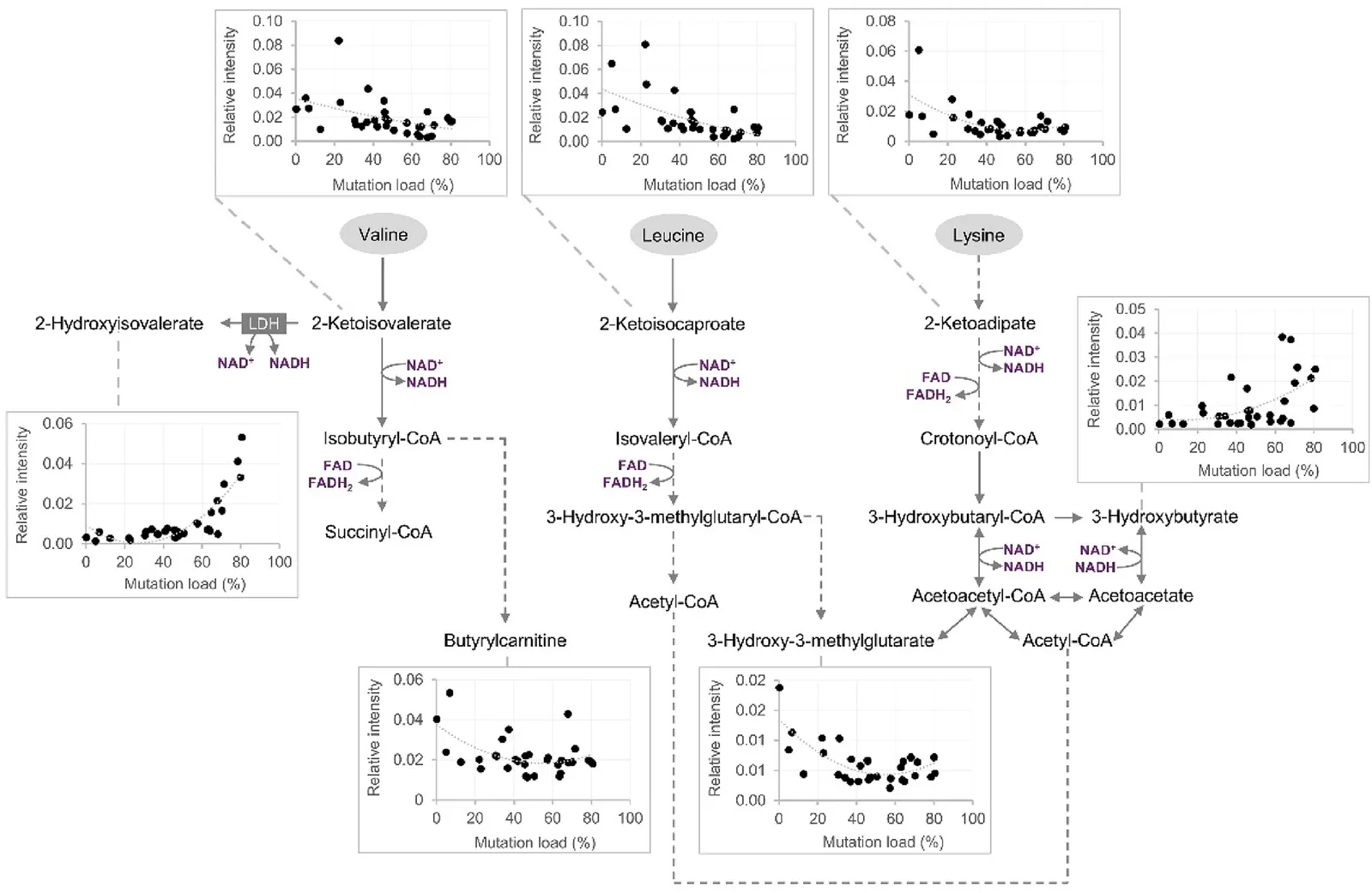
Intermediates of ketogenic and branched-chain amino acid metabolism correlate with mtDNA mutation load in Mito-mice. Scatterplots depict relative intensities (y-axis) of urinary metabolites in individual Mito-mice (*n* = 32; biologically independent samples) with varying levels of mtDNA mutation loads (x-axis). Metabolites that significantly correlated with mutation load are displayed in scatterplots (Pearson’s *r* > 0.4; FDR-*p* < 0.05). Isovaleryl-CoA, crotonoyl-CoA and acetoacetate as well as their conjugates were not detected. Solid and dashed arrows represent single- and multi-step reactions. Abbreviations: FDR, false discovery rate; LDH, lactate dehydrogenase

A metabolic signature associated with mtDNA mutation burden was evident in Mito-mice (Table 2), with 20 metabolites showing statistically significant associations (Pearson’s *r* > 0.4, FDR-adjusted *p* < 0.05). These included metabolites predominantly involved in glycolysis and the TCA cycle (Fig. 1), as well as pathways of ketogenic- and branched-chain amino acid metabolism (Fig. 2). Scatter plots for other significant correlations and metabolite ratios are given in Fig. S1. While adjustment for age and sex modestly attenuated correlation strengths for most metabolites, key associations remained robust. These findings were supported by both Pearson’s and Spearman’s analyses (Table S5), which yielded relatively consistent results.

Overall, two distinct response patterns emerged in relation to increasing mutation load. A subset of metabolites exhibited an approximately linear decline, while others exhibited a threshold-like trajectory — marked by minimal changes at low mutation loads (< 20%), gradual increases at intermediate levels (21–59%), and sharp elevations beyond ~ 60%. This latter pattern was most evident for 2-hydroxyisovalerate (2-HIVA), pyruvate, fumarate, malate, and 3-hydroxybutyrate. Notably, several of the declining metabolites appeared to plateau or exhibit a modest rebound at mutation loads above 60%, suggesting non-linear dynamics at this threshold point.

Among all measured features, 2-HIVA showed the strongest association with mutation load (Pearson’s *r* = 0.77; FDR-adjusted *p* = 8.43E-06), and related pathway metabolite ratios — 2-HIVA/2-ketoisovalerate (2-KIVA) and 2-KIVA/valine — also correlated robustly.

**Table 2:** Urinary metabolites and metabolite ratios significantly correlated with mtDNA mutation load in Mito-mice

Metabolites	Uncorrected			Corrected for sex and age		
	Pearson’s *r*	*p*-value	FDR-*p* value	Pearson’s *r*	*p*-value	FDR-*p* value
2-Hydroxyisovalerate (2-HIVA)	0.768	2.91E-07	8.43E-06	0.696	1.94E-05	4.08E-04
Pyruvate	0.686	1.49E-05	1.56E-04	0.563	1.19E-03	4.99E-03
Threonate	−0.649	5.83E-05	3.73E-04	−0.572	9.58E-04	4.82E-03
Fumarate	0.647	6.21E-05	3.73E-04	0.530	2.61E-03	7.69E-03
2-Ketoisocaproate	−0.642	7.54E-05	3.96E-04	−0.600	4.62E-04	3.88E-03
Lactate	0.636	9.08E-05	4.24E-04	0.547	1.77E-03	5.73E-03
Malate	0.613	1.89E-04	7.93E-04	0.485	6.65E-03	1.64E-02
Alanine	0.573	6.05E-04	1.82E-03	0.467	9.34E-03	2.12E-02
cis-Aconitate	−0.573	6.08E-04	1.82E-03	−0.512	3.84E-03	1.01E-02
Cystine	0.563	7.94E-04	2.22E-03	0.606	3.87E-04	3.88E-03
Succinate	−0.552	1.04E-03	2.74E-03	−0.585	6.78E-04	4.07E-03
3-Hydroxybutyrate	0.519	2.32E-03	5.73E-03	0.547	1.77E-03	5.73E-03
2-Ketoisovalerate (2-KIVA)	−0.496	3.85E-03	8.99E-03	−0.448	1.31E-02	2.34E-02
3-Hydroxy-3-methylglutarate	−0.485	4.90E-03	1.08E-02	−0.447	1.34E-02	2.34E-02
2-Ketoadipate	−0.479	5.59E-03	1.17E-02	−0.465	9.59E-03	2.12E-02
Arabinofuranose	0.466	7.17E-03	1.43E-02	0.527	2.75E-03	7.69E-03
2-Hydroxyglutarate	−0.447	1.03E-02	1.83E-02	−0.428	1.83E-02	3.08E-02
Ribitol	−0.446	1.04E-02	1.83E-02	−0.448	1.29E-02	2.34E-02
Phosphoglycerol	−0.422	1.62E-02	2.71E-02	−0.391	3.27E-02	5.29E-02
Rhamnose	−0.407	2.08E-02	3.36E-02	−0.331	7.44E-02	1.04E-01
Hexanoylglycine	−0.379	3.23E-02	5.03E-02	−0.557	1.37E-03	5.25E-03
Butyrylcarnitine	−0.364	4.08E-02	5.90E-02	−0.569	1.03E-03	4.82E-03
**Metabolite ratios**						
2-HIVA/2-KIVA	0.762	4.02E-07	8.43E-06	0.700	1.65E-05	4.08E-04
Fumarate/succinate	0.712	4.82E-06	6.74E-05	0.617	2.82E-04	3.88E-03
2-KIVA/valine	−0.655	4.68E-05	3.73E-04	−0.590	5.98E-04	4.07E-03
Malate/aspartate	0.600	2.80E-04	1.07E-03	0.451	1.24E-02	2.34E-02
Malate/fumarate	−0.581	4.94E-04	1.73E-03	−0.456	1.13E-02	2.34E-02
Lactate/pyruvate	0.460	8.14E-03	1.55E-02	0.337	6.86E-02	9.93E-02

### Conserved urinary metabolic alterations in m.3243 A > G patients and Mito-mice

**Fig. 3:**
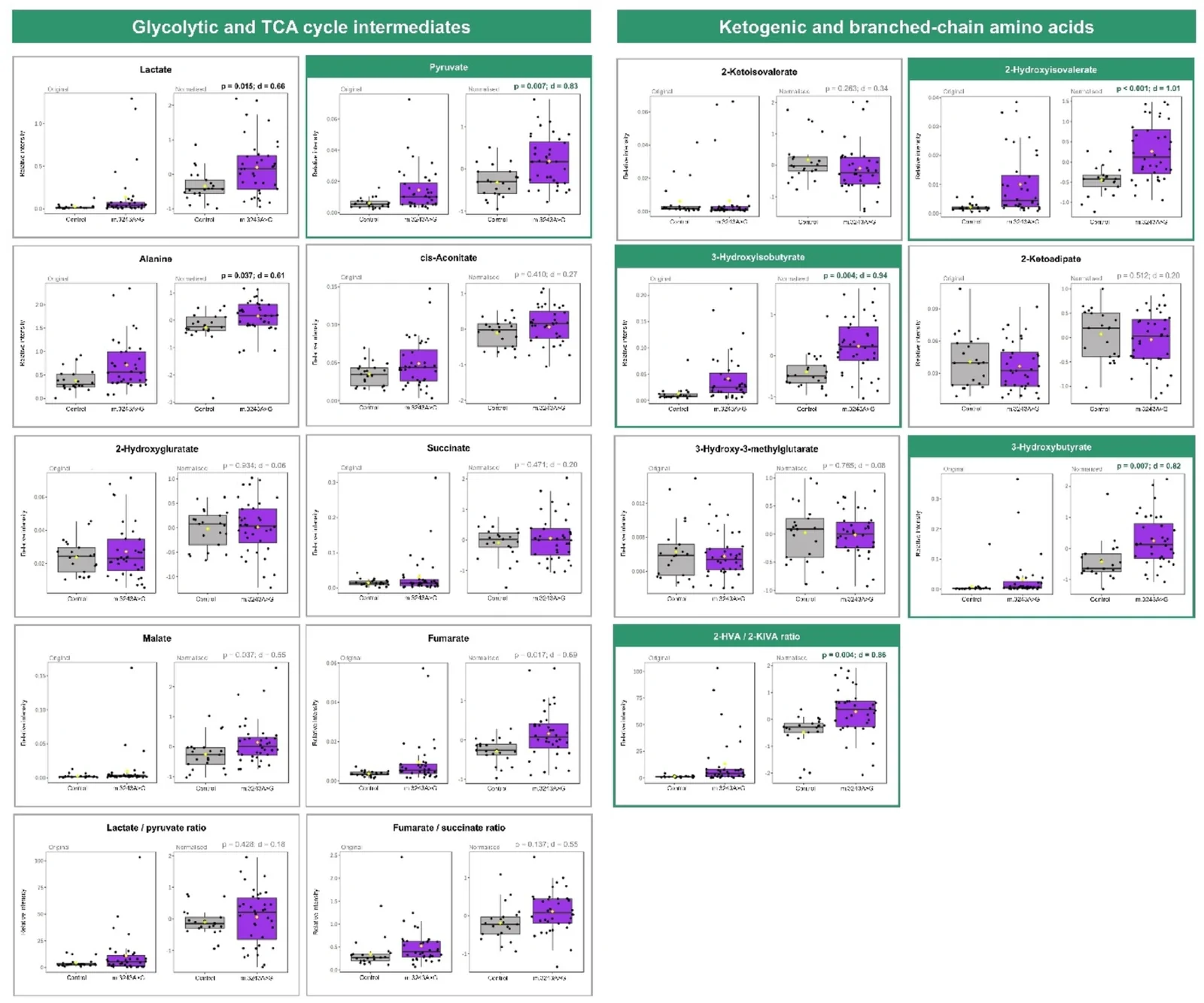
Urinary metabolites from energy and amino acid in m.3243 A > G patients and controls. Boxplots of metabolites involved in glycolysis, the TCA cycle, branched-chain- and ketogenic amino acid metabolism in controls (grey, *n* = 21) and m.3243 A > G patients (purple, *n* = 36), displayed before and after data normalisation (log transformation followed by pareto scaling). Each point represents an individual participant; yellow dots indicate group means and horizontal lines represent medians. Metabolite levels are expressed in relative intensity units. Statistical comparisons were conducted using the limma package with age and sex included as covariates. p-values were false discovery rate (FDR) corrected. Effect sizes (Cohen’s d) were calculated on log-transformed (but not pareto scaled) data to better reflect biological variance. Metabolites meeting both statistical and biological significance thresholds (FDR-adjusted *p* < 0.05 and d > 0.8) are outlined in green, with corresponding values shown in green text

To evaluate the translational relevance of the findings in Mito-mice, we assessed urinary metabolites from the same pathways in an adult patient cohort (*n* = 36) with the m.3243 A > G mutation (Fig. 3). Among these, twelve metabolites were significantly altered in patients compared to controls, of which ten remained significant after FDR correction. Adjustment for age and sex marginally increased the strength of several associations, indicating potential confounding. Several metabolites, including lactate, alanine, malate, and fumarate, exhibited medium effect sizes (Cohen’s d ≥ 0.5), while only four metabolites — 2-HIVA, 3-hydroxyisobutyrate, pyruvate, and 3-hydroxybutyrate — showed large effect sizes (Cohen’s d ≥ 0.8). Consistent with the mouse data, these metabolites were elevated in patient urine, with 2-HIVA exhibiting the strongest difference and the 2-HIVA/2-KIVA ratio also significantly altered.

Additionally, to investigate whether any of these metabolites reflect disease burden within the patient cohort, correlations with mutation load and clinical severity (NMDAS score) were assessed. An initial moderate positive correlation was observed between 2-HIVA and mutation load (Pearson’s *r* = 0.424, *p* = 0.010); however, this association was no longer significant after adjusting for age and sex. Furthermore, no significant relationship was observed between metabolites and NMDAS scores within the patient group.

### 2-Hydroxyisovalerate as a candidate biomarker for mtDNA-based disorders

**Fig. 4:**
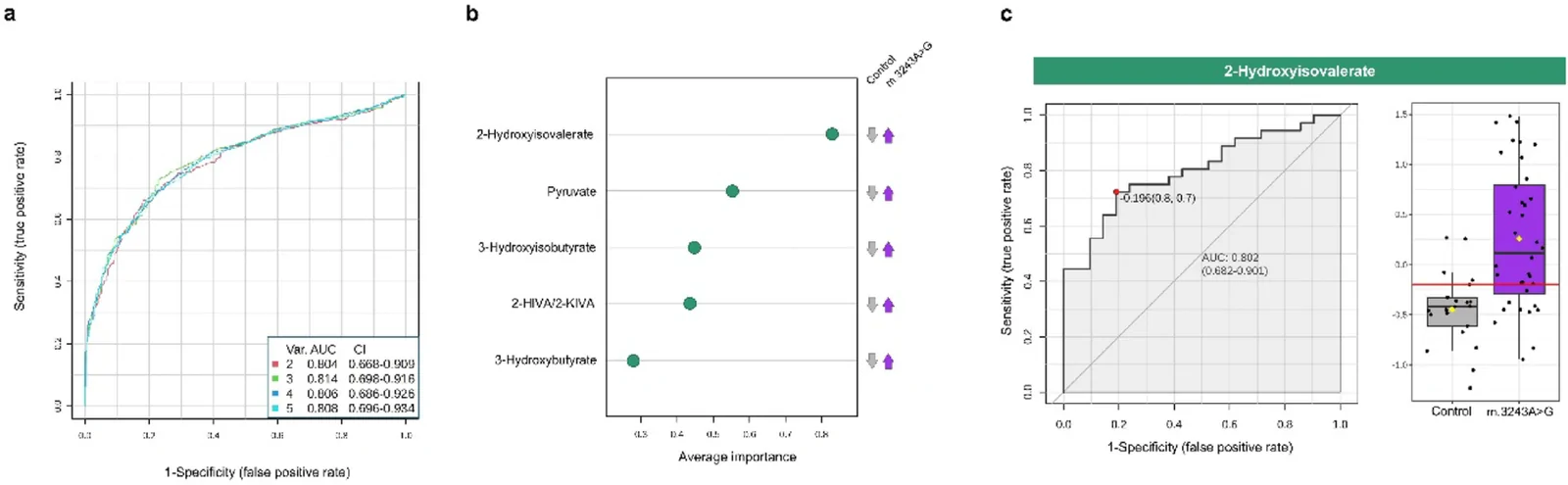
Discriminatory performance of urinary metabolites in distinguishing m.3243 A > G patients from controls. **a** Multivariate receiver operating characteristic (ROC) curves for four different biomarker models including the top discriminatory metabolites with their corresponding area under the curve (AUC) value and confidence interval (CI). **b** Average importance of selected metabolites in group classification models, with arrows indicating direction of change (grey = lower in controls, purple = higher in m.3243 A > G). 2-HIVA shows the highest importance. **c** Univariate ROC curve (left) and normalised boxplot (right) for 2-HIVA. The red dot and horizontal line show the optimal cutoff point with the associated sensitivity and specificity values

To assess the diagnostic utility of urinary metabolites in m.3243 A > G patients, multivariate ROC analysis (Fig. 4a) was performed incorporating the four most significantly altered individual metabolites (2-HIVA, 3-hydroxyisobutyrate, 3-hydroxybutyrate, pyruvate) and the top-ranked metabolite ratio (2-HIVA/2-KIVA). All four models incorporating 2–5 features demonstrated consistent discriminatory power, with mean cross-validated AUCs ranging from 0.80 to 0.81 and narrow 95% confidence intervals. Feature importance analysis (Fig. 4b) identified 2-HIVA as the most influential variable.

Consistently, univariate ROC analysis confirmed the strong standalone discriminatory power of 2-HIVA (Fig. 4c) with an AUC = 0.80 (*p* = 7.8E-05, fold change [FC] = 4.64), outperforming the other candidates: 3-hydroxyisobutyrate (AUC = 0.79, *p* = 0.00028, FC = 3.21), 2-HIVA/2-KIVA (AUC = 0.78, *p* = 0.0012, FC = 10.91), 3-hydroxybutyrate (AUC = 0.77, *p* = 0.0020, FC = 3.38), and pyruvate (AUC = 0.75, *p* = 0.0011, FC = 2.42). Thus, although the multivariate model of the entire biomarker panel (Fig. 4a, model 3) showed slightly higher accuracy, 2-HIVA alone showed robust potential as a standalone urinary biomarker.

## Discussion

### Systemic metabolic adaptions to progressive OXPHOS deficiency in Mito-mice

Mitochondrial dysfunction triggers a cascade of metabolic rewiring as cells attempt to maintain homeostasis. By examining urinary metabolite patterns across a gradient of mtDNA mutation load in Mito-mice, we observed correlations within TCA, redox, and amino acid pathways, reflecting key biochemical responses to increasing mitochondrial OXPHOS dysfunction.

The early linear decrease in TCA cycle intermediates — cis-aconitate, succinate, and the 2-ketoglutarate derivative 2-hydroxyglutarate — indicates a progressive reduction in carbon flux from acetyl-CoA through the TCA cycle as mtDNA mutation load increases. This likely reflects a slight downregulation of the TCA cycle as an initial, energy-conserving adaptation (Picard et al., 2014). However, beyond the ~ 60% mutation threshold, succinate levels plateaued or modestly rebounded, coinciding with sharp elevations in pyruvate (the end-product of glycolysis), fumarate (a CII product), and malate (a derivative of either fumarate or pyruvate). Pyruvate derivatives, lactate and alanine, followed a similar trend although lactate levels were variable, and alanine exhibited only modest elevation in most mice.

Increased urinary excretion of these metabolites is commonly associated with mitochondrial disorders and is largely driven by redox imbalance (Esterhuizen et al., 2017; Wolf & Smeitink, 2002; Rodenburg, 2011; Semeraro et al., 2018). Consistent with this mechanism, both the lactate/pyruvate ratio (Fig. S2c) — an indicator of cytosolic NADH/NAD^+^ balance (Munnich et al., 1996) — and the malate/aspartate ratio (Fig. S2f) — reflecting redox shuttle activity — exhibited a similar threshold-like increase in Mito-mice urine. Collectively, the abrupt changes at 60% mutation load are consistent with a reductive shift that may inhibit NAD⁺-dependent dehydrogenases and favours NADH-driven reductions, suggesting that rising mtDNA mutation load and worsening OXPHOS deficiency progressively disrupt the NADH/NAD⁺ balance, driving the observed metabolic abnormalities.

Such perturbations in NADH levels and TCA intermediates would induce a universal upregulation of OXPHOS complex subunits through retrograde signalling (Picard et al., 2014). However, in Mito-mice, only complex II or succinate dehydrogenase (SDH, EC 1.3.5.1) protein levels can be effectively increased, as all other OXPHOS complexes rely on mtDNA-encoded subunits and are impaired by the mtDNA deletion (Inoue et al., 2000). This exact outcome has been observed in cells harbouring the m.3243 A > G mutation, where transcript levels of all complexes rise, but only SDH exhibits a corresponding increase in protein abundance (Picard et al., 2014). Consistently, enhanced SDH activity has been reported in mouse models with mtDNA defects that impair CIV activity (Shimizu et al., 2014; Ishikawa et al., 2019). In Mito-mice, such an adaptation may increase fumarate and FADH₂ production, further aggravating redox imbalance due to dysfunction across the rest of the OXPHOS system. Supporting this, the urinary fumarate/succinate ratio (Fig. S2d) — an indicator of SDH activity (Kim et al., 2016) — correlated strongly with mutation load and displayed a marked shift at the 60% threshold. Despite the hypothesised increase in SDH expression and the presence of potential non-canonical sources of succinate, the observed plateau and modest rebound in succinate levels suggest that SDH activity remains limited — likely due to downstream respiratory chain bottlenecks, impaired FADH_2_ reoxidation, or restricted TCA cycle flux.

In addition to carbohydrate metabolism, amino acid pathways showed some of the strongest associations with mtDNA mutation load in Mito-mice. Notably, we observed a linear decrease in the urinary levels of 2-keto acid catabolites of valine (2-ketoisovalerate, 2-KIVA), leucine (2-ketoisocaproate), and lysine (2-ketoadipate), with increasing mutation load (Fig. 2). Since the catabolism of these amino acids requires NAD^+^ and FAD cofactors, perturbations in these pathways are another metabolic feature of OXPHOS dysfunction ascribed to redox imbalance (Esterhuizen et al., 2017). Although one would expect 2-keto acid accumulation in a highly reduced environment, these metabolites can be readily converted back to their cognate BCAA or reduced to 2-hydroxy acids (Liebich & Först, 1984; Prensky & Moser, 1966). The latter reaction seems prominent in Mito-mice as the levels of 2-hydroxyisovalerate (2-HIVA) increased with mutation load, whereas 2-KIVA, and another valine catabolite, butyrylcarnitine, decreased.

These observations suggest a shift in valine catabolism, resulting in the increased conversion of 2-KIVA to 2-HIVA, an NADH-dependent reaction catalysed by lactate dehydrogenase (LDH, EC 1.1.1.27) (Liebich & Först, 1984; Heemskerk et al., 2016). Although impaired activity of the branched-chain α-keto acid dehydrogenase complex might be expected to promote accumulation of branched-chain keto acids, our data instead suggest that these intermediates are preferentially diverted towards reduction to their corresponding hydroxy acids, particularly 2-HIVA, under conditions of increasing mitochondrial reductive stress. In support, the 2-KIVA/valine ratio (Fig. S2b) and 2-HIVA/2-KIVA ratio (Fig. S2a), displayed significant negative and positive correlations with mutation load, respectively. Since 2-HIVA and the 2-HIVA/2-KIVA ratio both increased markedly after ~ 60% mutation load, we propose that 2-HIVA production may serve to restore NAD^+^ levels in a highly reduced environment, similar to the production of lactate from pyruvate. To increase our confidence in these results, we used reference standards to confirm the identity of 2-HIVA and ensured that it was not a product of artificial conversion during sample preparation. Although 2-ketoacids of leucine, isoleucine, and lysine likely undergo a similar conversion to 2-hydroxy acids, these metabolites were not detected in our samples.

Additionally, levels of 3-hydroxy-3-methylglutarate decreased linearly with increasing mutation load. This metabolite can be derived from ketogenic amino acids and serves as a precursor for the ketone body 3-hydroxybutyrate (Pié et al., 2007). In Mito-mice, 3-hydroxybutyrate levels showed a positive correlation with mutation load, with a sharp increase beyond ~ 60% mutation load. The 3-hydroxybutyrate/acetoacetate ratio reflects the mitochondrial redox state since 3-hydroxybutyrate is reversibly formed from acetoacetate in an NADH-dependent reaction (Munnich et al., 1996). Although acetoacetate could not be quantified in this study, the marked elevations in 3-hydroxybutyrate at higher mutation loads (> 60%) further support a sudden rise in intramitochondrial redox imbalance and a shift towards ketone body production.

Together, these findings support a model in which initial compensatory responses to rising mutation burden are overwhelmed beyond ~ 60% heteroplasmy. The abrupt changes in multiple redox-sensitive metabolite ratios suggest a threshold effect, possibly mediated by nuclear-mitochondrial signalling cascades that fail to restore OXPHOS capacity.

### Conserved metabolic signatures in m.3243 A > G patients and the clinical relevance of 2-HIVA as a mechanistically anchored biomarker

Extending these observations to a clinical context, we explored urinary metabolites in patients harbouring the m.3243 A > G mtDNA mutation. Despite the cohort’s modest size (*n* = 36) and inherently greater biological variability compared to the controlled Mito-mice model, we observed consistent metabolic trends across both species.

Patients, diagnosed with classic MELAS or MIDD, exhibited significantly elevated urinary pyruvate, lactate, alanine, malate, and fumarate. Although most alterations were of modest magnitude, they mirrored that observed in Mito-mice with high mutation loads — consistent with advanced OXPHOS dysfunction and associated redox imbalance. While urinary pyruvate accumulation showed a large effect size in patients, its quantification in biofluids is technically challenging and may not accurately reflect in vivo concentrations. Oxaloacetate, which has a short half-life (7–14 h), spontaneously decarboxylates to form pyruvate (Fink et al., 2022; Yu & Sivitz, 2020). Considering the time taken to collect, transport, prepare, and analyse samples, measured urinary pyruvate likely represents a composite of both metabolites, complicating interpretation. Furthermore, the urinary lactate/pyruvate ratio (Fig S6) — a classic mitochondrial disease marker — did not differ significantly from controls. This is likely due to measurement instability in biofluids as lactate levels can vary greatly based on differences in sample collection and storage (Parikh et al., 2015). These limitations in the quantification of lactate and pyruvate highlight the need for more stable and specific urinary biomarkers of OXPHOS dysfunction.

The most pronounced urinary alterations in patients were observed in ketone body metabolism and valine catabolism, closely paralleling changes seen in Mito-mice. Elevated 3-hydroxybutyrate in patient urine suggests a compensatory shift toward ketone metabolism, likely reflecting OXPHOS dysfunction and impaired glucose oxidation. In parallel, patients showed markedly high levels of valine-derived catabolites, including 2-HIVA, the 2-HIVA/2-KIVA ratio, and the downstream metabolite 3-hydroxyisobutyrate. Elevated urinary 2-HIVA has been reported in various contexts of mitochondrial and metabolic impairment, including mtDNA depletion (Pajares et al., 2013), MIDD associated with the m.3243 A > G mutation (Esterhuizen et al., 2021; Chan et al., 2007), and disorders affecting BCAA metabolism such as lactic acidosis, ketoacidosis (Landaas & Jakobs, 1977), and maple syrup urine disease (Magni et al., 1994; Tomiko et al., 1983). Notably, 3-hydroxyisobutyrate has gained recognition as both a predictive biomarker for and a mechanistic contributor to insulin resistance and type 2 diabetes. By promoting fatty acid uptake into skeletal muscle, 3-hydroxyisobutyrate can lead to lipid accumulation, mitochondrial dysfunction, and oxidative stress — hallmarks of diabetic metabolic pathology. Elevated circulating levels of 3-hydroxyisobutyrate have been consistently associated with glucose intolerance and type 2 diabetes, suggesting that its increased urinary excretion in our cohort reflects not only underlying mitochondrial impairment but also systemic metabolic dysregulation driven by insulin resistance (Mardinoglu et al., 2018; Andersson-Hall et al., 2018; Nilsen et al., 2020; Dankel, 2023).

Beyond mechanistic relevance, our data also support the diagnostic value of these urinary metabolites and ratios in identifying individuals with mtDNA-based disorders. The strong classification performance in these metabolites highlights their promise as accessible, non-invasive biomarkers. Among these, 2-HIVA emerges as the most compelling candidate biomarker based on its (i) consistent elevation across species and mutation types, (ii) high standalone diagnostic performance (AUC > 0.8), and (iii) mechanistic plausibility as its NADH-dependent formation is consistent with redox shifts accompanying OXPHOS dysfunction. Although 2-HIVA is unlikely to be specific for mtDNA-based disorders, its consistent elevation across different mtDNA mutations and its mechanistic association with mitochondrial redox imbalance suggest that it may serve as a useful component of a broader biomarker panel rather than as a standalone diagnostic marker.

It is important to note, however, that while these metabolites reflect underlying bioenergetic impairment, their urinary concentrations did not correlate with mutation load or NMDAS scores after covariate adjustment in our patient cohort. This is consistent with the known non-linear relationship between heteroplasmy, tissue-specific vulnerability, and clinical severity in mitochondrial disease. However, plasma levels of some of these metabolites (pyruvate, lactate, alanine, and malate) were previously shown to correlate with disease severity in MELAS patients as measured by Karnofsky score (Sharma et al. 2021). This discrepancy underscores the need to consider biofluid-specific differences when evaluating candidate biomarkers.

In patients, urinary mtDNA mutation loads are known to exhibit greater variability compared to tissues and blood (Grady et al., 2018), while pharmacological treatment may have further obscured these associations. A substantial proportion of patients in our cohort were receiving insulin and/or anti-epileptic drugs (Table 1), both of which significantly impact systemic metabolism and renal handling of metabolites. Insulin is a central regulator of glucose, lipid, and protein metabolism (Bolli et al., 2021; Newsholme & Dimitriadis, 2001; Magkos et al., 2010) and its effects on metabolism may vary with dosing regimen, formulation, and patient-specific insulin sensitivity. Similarly, anti-epileptic drugs exhibit heterogeneous metabolic effects depending on the agent and dosage, with some directly impairing mitochondrial enzymes to varying degrees and others exhibiting protective effects on mitochondrial dynamics (Finsterer & Scorza, 2017; Stockburger et al., 2016; Kośmider et al., 2023). Beyond their systemic roles, both insulin (Nakamura et al., 2020) and anti-epileptic drugs (Anguissola et al., 2023; Hamed, 2017) also influence renal physiology through effects on proximal tubule transport, urine pH, or electrolyte balance, which can distort the relationship between systemic concentrations and urinary excretion patterns. Thus, metabolic heterogeneity driven by individual drug regimens, may largely contribute to the lack of correlation between urinary metabolites and clinical severity or mutation load in patients, despite clear associations in Mito-mice.

### Limitations and future directions

This study is limited by the restricted availability of Mito-mice, which precluded terminal tissue collection for complementary mechanistic analyses. Mutated mtDNA is selected against during oocyte development and is therefore lost within a few generations. To sustain a cohort of Mito-mice, new F0 transgenic mice would need to be generated every few generations, meaning Mito-mice are not easily bred and are not commercially available. The consequent scarcity of these mice and derived tissue meant that: (i) spot urine samples were collected as no metabolic cages were available at the facility where these mice are generated; and (ii) no tissue samples were available for follow-up studies to further investigate our initial findings. A further limitation is that renal dysfunction has been reported in Mito-mice with mutation loads exceeding 75% ΔmtDNA. Although only three animals in the present cohort exceeded this threshold, we cannot completely exclude a contribution of altered renal function to the urinary metabolite profile in these mice.

While urinary profiling is non-invasive and convenient, it may primarily reflect systemic or renal-specific effects rather than tissue-specific OXPHOS dysfunction. Nonetheless, the successful translation of key metabolic alterations from Mito-mice to human patients underscores the biological robustness and translational relevance of our findings.

Although these urinary metabolites effectively distinguished patients from controls, their lack of correlation with clinical severity or mutation load in the m.3243 A > G cohort limits their potential as longitudinal markers of disease progression. In contrast, several metabolites — particularly 2-HIVA — showed clear associations with mutation load in Mito-mice, suggesting that these metabolic changes are indeed linked to disease burden in a controlled biological context. This disconnection between mouse and patient data may arise from several uncontrolled sources of variability, including the known fluctuations in patient urinary mtDNA mutation loads, differences in diet, hydration status, and lifestyle, and the unavoidable continuation of medications that affect mitochondrial function and systemic metabolism. The latter may mask underlying mitochondrial signatures or exacerbate existing dysfunctions — complicating the interpretation of urinary metabolite profiles. A further limitation is that different urine normalisation strategies were used for the mouse and human datasets. This reflects the independent analytical workflows used for each cohort, with osmolality selected for the previously published human cohort (Esterhuizen et al., 2021) because creatinine was influenced by disease status, and creatinine used for the independently generated mouse cohort following validation during method development. Although this precludes direct comparison of absolute metabolite concentrations between species, the principal conclusions are based on the consistent direction of metabolite alterations observed across both cohorts.

Future studies may benefit from stratifying participants according to medication status or incorporating paired metabolite–hormone panels to better distinguish primary mitochondrial dysfunction from secondary drug effects. Because patients with mitochondrial disease could not ethically discontinue clinically indicated treatments, including insulin and anti-epileptic medication, and owing to the rarity of the disease, the limited cohort size and heterogeneity of treatment regimens, it was not possible to statistically account for the effects of individual medications in the present study. Consequently, a contribution of treatment to the observed urinary metabolomic profiles cannot be excluded. Larger and more controlled studies will therefore be essential to validate the diagnostic and prognostic utility of 2-HIVA and related metabolites. Longitudinal analyses including additional biofluids, such as plasma, will be particularly valuable in determining whether 2-HIVA reflects dynamic disease progression or responds to emerging redox-modulating therapies.

## Conclusion

Our findings provide compelling evidence for a distinct and conserved urinary metabolic signature associated with mtDNA-based OXPHOS dysfunction in both Mito-mice and m.3243 A > G patients. A pronounced metabolic shift occurring at ~ 60% mtDNA mutation load in Mito-mice marks a critical threshold beyond which compensatory mitochondrial adaptations seem to fail. Among the affected metabolites, 2-HIVA emerged as a robust and mechanistically informative biomarker of mitochondrial dysfunction, reflecting NADH-redox imbalance and disrupted valine catabolism. Its consistent elevation across species and mtDNA mutation types, along with the 2-HIVA/2-KIVA ratio, underscores its potential as a non-invasive biomarker for diagnosis, disease monitoring, and therapeutic response in mtDNA-based disorders. These findings support the integration of 2-HIVA and related ratios into urinary metabolite panels for clinical evaluation, where they may offer advantages over traditional markers such as lactate and the lactate/pyruvate ratio. Further longitudinal studies are warranted to validate their clinical utility and to determine their specificity across diverse genetic backgrounds, including other mtDNA mutations and nuclear-encoded mitochondrial disorders.

## Supplementary Information

Below is the link to the electronic supplementary material.

Supplementary Material 1

## Data Availability

The datasets generated during and/or analysed during the study are available from the corresponding author upon reasonable request.
