# Supplementary material for: Cross-species urinary metabolomics identifies 2-hydroxyisovalerate as a candidate biomarker for mtDNA-based disorders
Source: Metabolomics. 2026 Aug 20;22(5):140. doi: 10.1007/s11306-026-02518-1 (PMC13493467; doi:10.1007/s11306-026-02518-1)
Supplement: Supplementary file 1 [file 11306_2026_2518_MOESM1_ESM.docx]

Supplementary information

__________________________________________________________

Cross-species urinary metabolomics identifies 2-hydroxyisovalerate as a candidate biomarker for mtDNA-based disorders

Marianne Venter^a*^, Karin Terburgh^a*^, Jeremie Z. Lindeque^a^, Emi Ogasawara^b^, Mirian C.H. Janssen^c,d^, Jan A.M. Smeitink^c^, Kazuto Nakada^e^ and Roan Louw^a^*^[1]^*

^a^Mitochondria Research Laboratory, Biomedical and Molecular Metabolism Research (BioMMet), Faculty of Natural and Agricultural Sciences, North-West University, Potchefstroom, South Africa,

^b^Department of Biological Sciences, Graduate School of Science, Osaka University, Toyonaka, Osaka 560-0043, Japan,

^c^Department of Pediatrics, Radboud Center for Mitochondrial Medicine, Radboud University Medical Center, Nijmegen, Netherlands,

^d^Department of Internal Medicine, Radboud Center for Mitochondrial Medicine, Radboud University Medical Center, Nijmegen, Netherlands,

^e^Faculty of Life and Environmental Sciences, University of Tsukuba, Tsukuba, Japan,

* co-first authors

Corresponding author: Roan Louw. Email Roan.Louw@nwu.ac.za

**Table S1 | Gas chromatography oven temperature program**

| Parameter | Target temperature (°C) | Rate (°C/min) | Hold time (min) |
| --- | --- | --- | --- |
| Initial temperature: | 70 |  | 1 |
| 1^st^ temperature ramp | 120 | 7 | - |
| 2^nd^ temperature ramp | 230 | 10 | - |
| 3^rd^ temperature ramp | 300 | 13 | 1 |
| Return to initial | 70 |  | - |

**Table S2 | Liquid chromatography gradient elution program**

| Mouse urine | | | | | Human urine | | | | |
| --- | --- | --- | --- | --- | --- | --- | --- | --- | --- |
| Time (min) | % A | % B | Hold time (min) | Flow rate (ml/min) | Time (min) | %A | % B | Hold time (min) | Flow rate (ml/min) |
| Initial | 95 | 5 | 1 | 0.25 | Initial | 95 | 5 | 1 | 0.2 |
| 7 | 82 | 18 |  | 0.25 | 5 | 82 | 18 | 3 | 0.2 |
| 16 | 0 | 100 | 5 | 0.25 | 15 | 0 | 100 | 5 | 0.3 |
| 24 | 95 | 5 | 24 | 0.25 | 23 | 95 | 5 |  | 0.2 |
| Post run | 95 | 5 | 10 | 0.25 | Post run | 95 | 5 | 10 | 0.2 |

A= water (0.1% formic acid), B= acetonitrile (0.1% formic acid).

**Table S3 | MRM conditions for compounds measured via LC–MS/MS in Mito-mice urine**

| Compound | Precursor ion (m/z) | Product ion (m/z) | F (V) | CE (V) |
| --- | --- | --- | --- | --- |
| Amino acids and derivatives | | | | |
| 1-Methyl-L-Histidine | 226.4 | 124.2 | 120 | 15 |
| 3-Alanine | 146.2 | 72.1 | 90 | 10 |
| 3-Hydroxykynurenine | 281.2 | 152.1 | 108 | 12 |
| 3-Aminoisobutyric acid | 160.1 | 30.1 | 100 | 15 |
| 3-Methyl-L-Histidine | 226.4 | 96.2 | 130 | 25 |
| 4-Aminobutyric acid | 160.1 | 87.1 | 79 | 8 |
| 4-Hydroxy-L-proline | 188.1 | 68.1 | 120 | 35 |
| 5-Hydroxy-L-lysine | 219.4 | 82.2 | 100 | 20 |
| Creatine | 188.1 | 90.1 | 104 | 16 |
| Kynurenine | 265.4 | 136 | 120 | 10 |
| L-2-Aminoadipic acid | 274.2 | 98.1 | 113 | 24 |
| L-Alanine / L-Sarcosine | 146.1 | 44.2 | 80 | 15 |
| L-Arginine | 231.2 | 70.1 | 103 | 40 |
| L-Arginine-*d_4_* | 235.2 | 74.2 | 108 | 32 |
| L-Asparagine | 189.1 | 144.1 | 89 | 8 |
| L-Asparagine-^13^C_4_,^15^N_2_ | 195.2 | 148.1 | 89 | 8 |
| L-Aspartic acid | 246.2 | 144.1 | 98 | 12 |
| L-Citrulline | 232.2 | 70.1 | 89 | 32 |
| L-Citrulline-d_4_ | 236.2 | 74.1 | 108 | 28 |
| L-Cystathionine | 335.2 | 190.1 | 127 | 16 |
| L-Cystine | 353.0 | 130.0 | 118 | 16 |
| L-Glutamic acid | 260.2 | 84.1 | 89 | 24 |
| L-Glutamine-^13^C_5_,^15^N_2_ | 210.2 | 89.1 | 89 | 20 |
| L-Glycine | 132.1 | 76.1 | 65 | 4 |
| L-Histidine | 212.1 | 110.1 | 89 | 16 |
| L-Isoleucine / L-Leucine | 188.2 | 86.2 | 89 | 8 |
| L-Isoleucine-d_10_ | 198.2 | 96.2 | 108 | 12 |
| L-Isothreonine | 176.1 | 74.1 | 94 | 12 |
| L-Lysine / L-Glutamine | 203.1 | 84.1 | 89 | 20 |
| L-Lysine-d_4_ | 207.2 | 88.2 | 118 | 16 |
| L-Methionine | 206.1 | 104.1 | 94 | 8 |
| L-Ornithine | 189.2 | 70.2 | 74 | 20 |
| L-Phenylalanine | 222.2 | 120.1 | 108 | 16 |
| L-Phenylalanine-(ring-d_5_) | 227.2 | 125.1 | 108 | 16 |
| L-Pipecolic acid | 186.1 | 84.1 | 108 | 16 |
| L-Proline | 172.1 | 70.2 | 94 | 20 |
| L-Pyroglutamic acid | 186.1 | 84.1 | 108 | 16 |
| L-Serine | 162.1 | 60.1 | 94 | 12 |
| L-Threonine | 176.1 | 158.1 | 94 | 5 |
| L-Tryptophan | 261.2 | 244.1 | 94 | 8 |
| L-Tyrosine | 238.2 | 136.1 | 94 | 12 |
| L-Valine | 174.2 | 72.2 | 89 | 12 |
| L-Valine-d_8_ | 182.2 | 80.2 | 103 | 12 |
| *N, N, N*-Trimethylglycine | 174.2 | 58.2 | 130 | 45 |
| *N, N*-Dimethylglycine | 160.3 | 58.2 | 90 | 20 |
| *N, N*-Dimethyl-L-phenylalanine | 250.1 | 148.1 | 103 | 20 |
| *N*-Acetylaspartic acid | 288.2 | 144.1 | 98 | 16 |
| *N*-Acetylglutamic acid | 302.2 | 186.1 | 113 | 12 |
| Taurine | 126.0 | 107.9 | 93 | 10 |
| Acylcarnitines | | | | |
| Acetyl-L-carnitine (C2) | 260.2 | 85.1 | 122 | 24 |
| Acetyl-L-carnitine-d_3_ | 263.2 | 85.1 | 127 | 20 |
| Butyryl-L-carnitine (C4) | 288.2 | 85.1 | 110 | 20 |
| L-Carnitine (C0) | 218.2 | 103.0 | 132 | 16 |
| L-Carnitine-(methyl-d_3_) | 221.2 | 103.1 | 127 | 16 |
| Decanoyl-L-carnitine (C10) | 372.3 | 85.1 | 125 | 28 |
| Dodecanoyl-L-carnitine (C12) | 400.3 | 85.1 | 155 | 28 |
| Dodecanoyl-L-carnitine-*d_3_* | 403.4 | 85.1 | 160 | 32 |
| Hexadecanoyl-L-carnitine (C16) | 456.4 | 85.1 | 160 | 36 |
| Hexanoyl-L-carnitine (C6) | 316.2 | 85.1 | 137 | 24 |
| Isovaleryl-L-carnitine (C5) | 302.2 | 85.1 | 105 | 24 |
| Octadecanoyl-L-carnitine (C18) | 484.0 | 85.1 | 155 | 36 |
| Octadecanoyl-L-carnitine-d_3_ | 487.5 | 85.1 | 160 | 36 |
| Octanoyl-L-carnitine (C8) | 344.3 | 85.1 | 115 | 28 |
| Octanoyl-L-carnitine-d_3_ | 347.3 | 85.1 | 136 | 24 |
| Propionyl-L-carnitine (C3) | 274.2 | 85.1 | 127 | 20 |
| Tetradecanoyl-L-carnitine (C14) | 428.4 | 85.1 | 150 | 28 |

**Table S4 | Targeted data extraction for compounds measured via GC–TOF–MS in Mito-mice urine**

| **Metabolite** | **Ion** | **RT** | **ID level** |
| --- | --- | --- | --- |
| 2,3-Dihydroxybutanoate | 117 | 655.35 | 3 |
| 2-Hydroxyglutarate | 129 | 828.5 | 1 |
| 2-Hydroxyisovalerate | 145 | 452.1 | 1 |
| 2-Ketoadipate | 55 | 877.3 | 3 |
| 2-Ketoisocaproate | 200/216 | 489 | 3 |
| 2-Ketoisovalerate | 89 | 452.2 | 1 |
| 3-Hydroxy-3-methylglutarate | 109 | 850.5 | 3 |
| 3-Hydroxybutyrate | 191/233 | 442.25 | 1 |
| 3-Phenylbutyrate (IS) | 118 | 704.25 | 1 |
| 4-Nitrophenyl-alfa-galacturonide | 73 | 1289.3/1400.3 | 3 |
| cis-Aconitate | 229 | 945 | 1 |
| Alanine | 116 | 380.25/406.15 | 1 |
| Arabinofuranose | 217 | 1253.65/1294.35 | 2 |
| Fumarate | 245 | 634.2 | 1 |
| Galactose | 205 | 1094.7 | 2 |
| Gluconate | 103/147 | 1139.55 | 2 |
| Gulonate | 81 | 1066.65 |  |
| Hexanoylglycine | 158/230 | 848.65 | 1 |
| Hydroxylamine | 133 | 401.85 | 3 |
| Lactate | 117/191 | 333 | 1 |
| Malate | 55 | 764.35 | 1 |
| Oxalate | 147 | 395.3 | 1 |
| Phosphoglycerol | 299 | 964.95 | 3 |
| Proline | 142 | 588.55 | 1 |
| Pyruvate | 207/174 | 305.7 | 1 |
| Rhamnose | 117 | 955.65 | 2 |
| Ribitol | 103 | 1058.35 | 2 |
| Succinate | 247 | 597.6 | 1 |
| Threonate | 292 | 831.7 | 3 |
| Tropic acid (IS) | 118 | 829.85 | 1 |
| Uracil | 241 | 619.05 | 1 |
| Urate | 441/456 | 1159.3 | 1 |

Identification (ID) levels were assigned according to the classification system of Schymanski *et al.* (2014), i.e., level 1 = confirmed structures (reference standard); level 2 = probable structure (library spectrum match); level 3 = putative candidate (structure, substituent, class). Abbreviation: IS, internal standard.

**Table S5 | Spearman’s rho for urinary metabolites and metabolite ratios significantly correlated with mtDNA mutation load in Mito-mice**

| **Spearman** | **Uncorrected** | | | **Corrected for sex and age** | | |
| --- | --- | --- | --- | --- | --- | --- |
|  | Spearman's ρ | p-value | FDR | Spearman's ρ | p-value | FDR |
| 2-Hydroxyisovalerate | 0.786 | 9.66E-07 | 2.10E-05 | 0.713 | 9.74E-06 | 2.04E-04 |
| Pyruvate | 0.708 | 1.08E-05 | 1.13E-04 | 0.588 | 6.32E-04 | 2.79E-03 |
| 2-Ketoisocaproate | -0.657 | 6.73E-05 | 4.71E-04 | -0.555 | 1.47E-03 | 5.40E-03 |
| Fumarate | 0.650 | 8.45E-05 | 5.04E-04 | 0.538 | 2.17E-03 | 6.52E-03 |
| cis-Aconitate | -0.644 | 1.02E-04 | 5.04E-04 | -0.601 | 4.42E-04 | 2.65E-03 |
| Lactate | 0.638 | 1.23E-04 | 5.15E-04 | 0.592 | 5.68E-04 | 2.79E-03 |
| Alanine | 0.623 | 1.91E-04 | 7.30E-04 | 0.586 | 6.65E-04 | 2.79E-03 |
| Threonate | -0.620 | 2.08E-04 | 7.30E-04 | -0.528 | 2.74E-03 | 7.41E-03 |
| Malate | 0.603 | 3.37E-04 | 1.01E-03 | 0.484 | 6.77E-03 | 1.50E-02 |
| Cystine | 0.566 | 8.91E-04 | 2.49E-03 | 0.610 | 3.48E-04 | 2.43E-03 |
| Succinate | -0.510 | 3.21E-03 | 8.44E-03 | -0.517 | 3.47E-03 | 8.58E-03 |
| 3-Hydroxybutyrate | 0.505 | 3.61E-03 | 8.91E-03 | 0.626 | 2.18E-04 | 1.83E-03 |
| 2-Ketoisovalerate | -0.457 | 9.08E-03 | 2.03E-02 | -0.363 | 4.84E-02 | 7.26E-02 |
| Rhamnose | -0.444 | 1.17E-02 | 2.45E-02 | -0.388 | 3.42E-02 | 5.87E-02 |
| Phosphoglycerol | -0.437 | 1.30E-02 | 2.60E-02 | -0.442 | 1.44E-02 | 2.75E-02 |
| Ribitol | -0.428 | 1.52E-02 | 2.90E-02 | -0.372 | 4.30E-02 | 6.68E-02 |
| 2,3-Dihydroxybutanoate | -0.421 | 1.71E-02 | 3.12E-02 | -0.379 | 3.89E-02 | 6.28E-02 |
| Arabinofuranose | 0.403 | 2.29E-02 | 4.00E-02 | 0.512 | 3.86E-03 | 9.02E-03 |
| Butyrylcarnitine | -0.316 | 7.85E-02 | 1.14E-01 | -0.526 | 2.82E-03 | 7.41E-03 |
| Hexanoylglycine | -0.275 | 1.28E-01 | 1.58E-01 | -0.553 | 1.54E-03 | 5.40E-03 |
| Hexanoylcarnitine | -0.375 | 3.54E-02 | 5.72E-02 | -0.395 | 3.06E-02 | 5.59E-02 |
| 2-Hydroxyglutarate | -0.331 | 6.44E-02 | 1.00E-01 | -0.278 | 1.36E-01 | 1.98E-01 |
| 2-Ketoadipate | -0.318 | 7.67E-02 | 1.14E-01 | -0.256 | 1.72E-01 | 2.32E-01 |
| 3-Hydroxy-3-methylglutarate | -0.307 | 8.79E-02 | 1.23E-01 | -0.253 | 1.76E-01 | 2.32E-01 |
| Metabolite ratios |  |  |  |  |  |  |
| 2-HIVA/2-KIVA | 0.784 | 9.98E-07 | 2.10E-05 | 0.744 | 2.50E-06 | 1.05E-04 |
| Fumarate/Succinate | 0.725 | 5.61E-06 | 7.85E-05 | 0.640 | 1.39E-04 | 1.83E-03 |
| 2KIVA/Valine | -0.701 | 1.42E-05 | 1.19E-04 | -0.628 | 2.05E-04 | 1.83E-03 |
| Malate/Fumarate | -0.642 | 1.08E-04 | 5.04E-04 | -0.546 | 1.81E-03 | 5.84E-03 |
| Malate/Aspartate | 0.606 | 3.11E-04 | 1.00E-03 | 0.464 | 9.85E-03 | 2.07E-02 |
| Lactate/Pyruvate | 0.457 | 9.20E-03 | 2.03E-02 | 0.386 | 3.50E-02 | 5.87E-02 |

**Limma results**

| **No adjustment** | logFC | AveExpr | t | P.Value | adj.P.Val | B |  | **Adjust for sex and age** | logFC | FC | AveExpr | t | Cohens d | P.Value | adj.P.Val | B |
| --- | --- | --- | --- | --- | --- | --- | --- | --- | --- | --- | --- | --- | --- | --- | --- | --- |
| 2-Hydroxy-isovaleric acid | 0.70 | -1.55E-16 | 4.27 | 5.73E-05 | 1.72E-03 | 1.59 |  | 2-Hydroxy-isovaleric acid | 0.73 | 1.66 | -1.55E-16 | 4.58 | 1.26 | 1.91E-05 | 5.73E-04 | 2.59 |
| 3-Hydroxy(iso)butyric acid | 0.63 | -1.48E-16 | 3.86 | 2.42E-04 | 3.62E-03 | 0.25 |  | 3-Hydroxy(iso)butyric acid | 0.62 | 1.54 | -1.48E-16 | 3.74 | 1.03 | 3.63E-04 | 4.10E-03 | -0.17 |
| 2-HIVA/2-KIVA | 0.77 | -1.21E-17 | 3.63 | 5.26E-04 | 5.26E-03 | -0.47 |  | 2-HIVA/2-KIVA | 0.80 | 1.74 | -1.21E-17 | 3.71 | 1.02 | 4.10E-04 | 4.10E-03 | -0.28 |
| 3-Hydroxybutyric acid | 0.67 | 7.64E-17 | 3.41 | 1.07E-03 | 8.03E-03 | -1.12 |  | 3-Hydroxybutyric acid | 0.69 | 1.61 | 7.64E-17 | 3.40 | 0.93 | 1.11E-03 | 6.80E-03 | -1.20 |
| Pyruvate | 0.50 | -5.69E-17 | 3.33 | 1.34E-03 | 8.05E-03 | -1.33 |  | Pyruvate | 0.51 | 1.43 | -5.69E-17 | 3.39 | 0.93 | 1.13E-03 | 6.80E-03 | -1.22 |
| Fumarate | 0.45 | 3.84E-16 | 2.91 | 4.79E-03 | 2.11E-02 | -2.48 |  | Lactate | 0.58 | 1.50 | -1.39E-16 | 3.08 | 0.85 | 2.96E-03 | 1.48E-02 | -2.10 |
| Lactate | 0.54 | -1.39E-16 | 2.90 | 4.92E-03 | 2.11E-02 | -2.51 |  | Fumarate | 0.47 | 1.38 | 3.84E-16 | 2.97 | 0.82 | 4.00E-03 | 1.72E-02 | -2.37 |
| Oxalate | 0.42 | -3.62E-17 | 2.77 | 7.05E-03 | 2.64E-02 | -2.83 |  | Proline | 0.65 | 1.56 | 2.92E-18 | 2.89 | 0.79 | 5.14E-03 | 1.93E-02 | -2.60 |
| Proline | 0.60 | 2.92E-18 | 2.62 | 1.06E-02 | 3.53E-02 | -3.19 |  | Oxalate | 0.43 | 1.35 | -3.62E-17 | 2.81 | 0.77 | 6.33E-03 | 2.11E-02 | -2.79 |
| Alanine | 0.41 | 4.29E-17 | 2.49 | 1.51E-02 | 4.53E-02 | -3.50 |  | Malate | 0.45 | 1.37 | -1.02E-16 | 2.54 | 0.70 | 1.33E-02 | 3.70E-02 | -3.45 |
| Malate | 0.40 | -1.02E-16 | 2.24 | 2.84E-02 | 7.74E-02 | -4.04 |  | Alanine | 0.42 | 1.34 | 4.29E-17 | 2.53 | 0.69 | 1.36E-02 | 3.70E-02 | -3.47 |
| Uric acid 4TMS | 0.31 | -1.70E-17 | 2.06 | 4.31E-02 | 1.01E-01 | -4.40 |  | 2,3-Dihydroxybutanoic acid | 0.39 | 1.31 | 9.47E-17 | 2.02 | 0.55 | 4.74E-02 | 1.13E-01 | -4.54 |
| Fumarate/Succinate | 0.31 | 4.85E-17 | 2.04 | 4.48E-02 | 1.01E-01 | -4.43 |  | *Uric acid 4TMS* | 0.29 | 1.23 | -1.70E-17 | 2.00 | 0.55 | 4.90E-02 | 1.13E-01 | -4.57 |
| 2,3-Dihydroxybutanoic acid | 0.38 | 9.47E-17 | 2.02 | 4.70E-02 | 1.01E-01 | -4.47 |  | Fumarate/Succinate | 0.29 | 1.22 | 4.85E-17 | 1.88 | 0.52 | 6.38E-02 | 1.37E-01 | -4.79 |
| Sarcosine | -0.23 | -1.51E-17 | -1.88 | 6.43E-02 | 1.29E-01 | -4.73 |  | Sarcosine | -0.23 | 0.85 | -1.51E-17 | -1.79 | -0.49 | 7.74E-02 | 1.50E-01 | -4.95 |
| Ribitol | 0.36 | 2.24E-17 | 1.73 | 8.80E-02 | 1.65E-01 | -4.99 |  | Ribitol | 0.38 | 1.30 | 2.24E-17 | 1.78 | 0.49 | 7.98E-02 | 1.50E-01 | -4.97 |
| Phosphoglycerol | 0.31 | 2.75E-16 | 1.62 | 1.10E-01 | 1.94E-01 | -5.16 |  | 3-Hydroxyisovaleric acid | 0.26 | 1.20 | 5.45E-18 | 1.60 | 0.44 | 1.14E-01 | 2.01E-01 | -5.26 |
| 3-Hydroxyisovaleric acid | 0.25 | 5.45E-18 | 1.58 | 1.18E-01 | 1.96E-01 | -5.22 |  | 2-Keto-isovaleric acid | -0.29 | 0.82 | -2.83E-16 | -1.43 | -0.39 | 1.57E-01 | 2.62E-01 | -5.51 |
| 2-Keto-isovaleric acid | -0.27 | -2.83E-16 | -1.37 | 1.76E-01 | 2.78E-01 | -5.52 |  | Phosphoglycerol | 0.24 | 1.18 | 2.75E-16 | 1.27 | 0.35 | 2.08E-01 | 3.29E-01 | -5.71 |
| Aconitic acid | 0.17 | 1.18E-16 | 1.09 | 2.78E-01 | 4.17E-01 | -5.85 |  | Aconitic acid | 0.17 | 1.13 | 1.18E-16 | 1.10 | 0.30 | 2.73E-01 | 4.10E-01 | -5.91 |
| Succinate | 0.15 | 4.60E-17 | 0.81 | 4.18E-01 | 5.81E-01 | -6.11 |  | Succinate | 0.18 | 1.13 | 4.60E-17 | 0.98 | 0.27 | 3.30E-01 | 4.71E-01 | -6.03 |
| Lac/Pyr | 0.16 | 3.09E-17 | 0.80 | 4.26E-01 | 5.81E-01 | -6.12 |  | Lac/Pyr | 0.19 | 1.14 | 3.09E-17 | 0.93 | 0.26 | 3.54E-01 | 4.82E-01 | -6.08 |
| 2-Keto-adipic acid | -0.11 | -1.82E-16 | -0.73 | 4.65E-01 | 6.06E-01 | -6.17 |  | 2-Keto-adipic acid | -0.13 | 0.91 | -1.82E-16 | -0.86 | -0.24 | 3.92E-01 | 5.12E-01 | -6.14 |
| Threonic acid | 0.08 | 5.40E-17 | 0.51 | 6.15E-01 | 7.68E-01 | -6.31 |  | Rhamnose | -0.07 | 0.95 | 1.73E-16 | -0.47 | -0.13 | 6.43E-01 | 7.65E-01 | -6.40 |
| Uracil | 0.05 | 1.55E-16 | 0.35 | 7.30E-01 | 8.48E-01 | -6.37 |  | Threonic acid | 0.07 | 1.05 | 5.40E-17 | 0.45 | 0.12 | 6.54E-01 | 7.65E-01 | -6.40 |
| Gulonic acid | 0.06 | -1.47E-16 | 0.34 | 7.35E-01 | 8.48E-01 | -6.38 |  | 3-Hydroxy-3-methylglutaric acid | -0.06 | 0.96 | -1.17E-16 | -0.44 | -0.12 | 6.63E-01 | 7.65E-01 | -6.41 |
| 3-Hydroxy-3-methylglutaric acid | -0.04 | -1.17E-16 | -0.30 | 7.67E-01 | 8.52E-01 | -6.39 |  | Malate/Fumarate | 0.06 | 1.04 | -9.21E-18 | 0.33 | 0.09 | 7.45E-01 | 8.05E-01 | -6.45 |
| 2-Hydroxyglutaric acid | 0.03 | 1.40E-16 | 0.23 | 8.16E-01 | 8.74E-01 | -6.41 |  | Uracil | 0.05 | 1.03 | 1.55E-16 | 0.32 | 0.09 | 7.51E-01 | 8.05E-01 | -6.45 |
| Rhamnose | -0.03 | 1.73E-16 | -0.19 | 8.51E-01 | 8.81E-01 | -6.41 |  | Gulonic acid | 0.04 | 1.03 | -1.47E-16 | 0.23 | 0.06 | 8.21E-01 | 8.49E-01 | -6.48 |
| Malate/Fumarate | 0.02 | -9.21E-18 | 0.09 | 9.28E-01 | 9.28E-01 | -6.43 |  | 2-Hydroxyglutaric acid | 0.01 | 1.01 | 1.40E-16 | 0.08 | 0.02 | 9.34E-01 | 9.34E-01 | -6.50 |

**Fig. S1 | Other significant correlations between mutation load and metabolite levels in Mito-mice.** Scatter plots depict relative intensities (y-axis) of urinary metabolites in individual mito-mice (n = 32) with varying levels of mtDNA mutation loads (x-axis). Pearson’s r values are displayed on graphs.

**Fig. S2 | Metabolite ratios that correlate with mutation load in Mito-mice.** Scatter plots depict relative intensities (y-axis) of urinary metabolite ratios in individual mito-mice (n = 32) with varying levels of mtDNA mutation loads (x-axis). Pearson’s r values are displayed on graphs. Abbreviations: 2-HIVA, 2-hydroxyisovalerate; 2-KIVA, 2 -ketoisovalerate.
